# A recent deep earthquake doublet in light of long-term evolution of Nazca subduction

**DOI:** 10.1038/srep45153

**Published:** 2017-03-31

**Authors:** J. Zahradník, H. Čížková, C. R. Bina, E. Sokos, J. Janský, H. Tavera, J. Carvalho

**Affiliations:** 1 Charles University, Prague, Czech Republic; 2 Northwestern University, USA; 3 University of Patras, Greece; 4 Geophysical Institute of Peru, Lima, Peru; 5 Seismological Observatory, University of Brasilia, Brazil

## Abstract

Earthquake faulting at ~600 km depth remains puzzling. Here we present a new kinematic interpretation of two Mw7.6 earthquakes of November 24, 2015. In contrast to teleseismic analysis of this doublet, we use regional seismic data providing robust two-point source models, further validated by regional back-projection and rupture-stop analysis. The doublet represents segmented rupture of a ∼30-year gap in a narrow, deep fault zone, fully consistent with the stress field derived from neighbouring 1976–2015 earthquakes. Seismic observations are interpreted using a geodynamic model of regional subduction, incorporating realistic rheology and major phase transitions, yielding a model slab that is nearly vertical in the deep-earthquake zone but stagnant below 660 km, consistent with tomographic imaging. Geodynamically modelled stresses match the seismically inferred stress field, where the steeply down-dip orientation of compressive stress axes at ∼600 km arises from combined viscous and buoyant forces resisting slab penetration into the lower mantle and deformation associated with slab buckling and stagnation. Observed fault-rupture geometry, demonstrated likelihood of seismic triggering, and high model temperatures in young subducted lithosphere, together favour nanometric crystallisation (and associated grain-boundary sliding) attending high-pressure dehydration as a likely seismogenic mechanism, unless a segment of much older lithosphere is present at depth.

Ongoing debate over seismogenic mechanisms of deep-focus earthquakes^1^ revolves around transformational faulting^2^,^3^, thermal shear instabilities^4^,^5^, and dehydration embrittlement^6^. Here we report new seismic source analyses and associated geodynamic modelling to better constrain the most plausible mechanism behind a recent South American deep-focus doublet. An enigmatic earthquake ‘nest’ exists close the Peru-Brazil border at latitudes 6°–12° S. Approximately 20 deep-focus events of Mw > 6 (GCMT^7^,^8^ catalogue 1976–2015) form a narrow belt delineating a deep fault zone, ∼400** **km long and striking ∼160° (Fig. 1a), with depths confined (in 70% of cases) to a 30-km narrow range (590–620** **km). Why such well-expressed, spatially limited seismicity, and how does it relate to ongoing Nazca plate subduction beneath South America at 7–8** **cm/year^9^,^10^,^11^,^12^,^13^? The so-called thermal parameter^14^ of ∼1300–2000** **km ranks this subduction as ‘warm’, but the slab geometry^15^ is only weakly constrained by seismicity, because the depths 200–600** **km are aseismic. Below 650** **km, an almost horizontal stagnant slab has been imaged by seismic tomography^16^. To better understand the physics of Peru-Brazil subduction, we investigate fault processes of the most recent strong earthquakes, in the context of neighbouring seismicity. We derive a stress field that is successfully explained by a dynamical model of ∼100-My slab evolution consistent with tomography. Finally, we combine seismic source properties (rupture geometry and velocities, seismic triggering) and geodynamic temperature estimates to compare competing hypotheses of seismogenic mechanism.

## Seismic Observations

**Figure 1:**
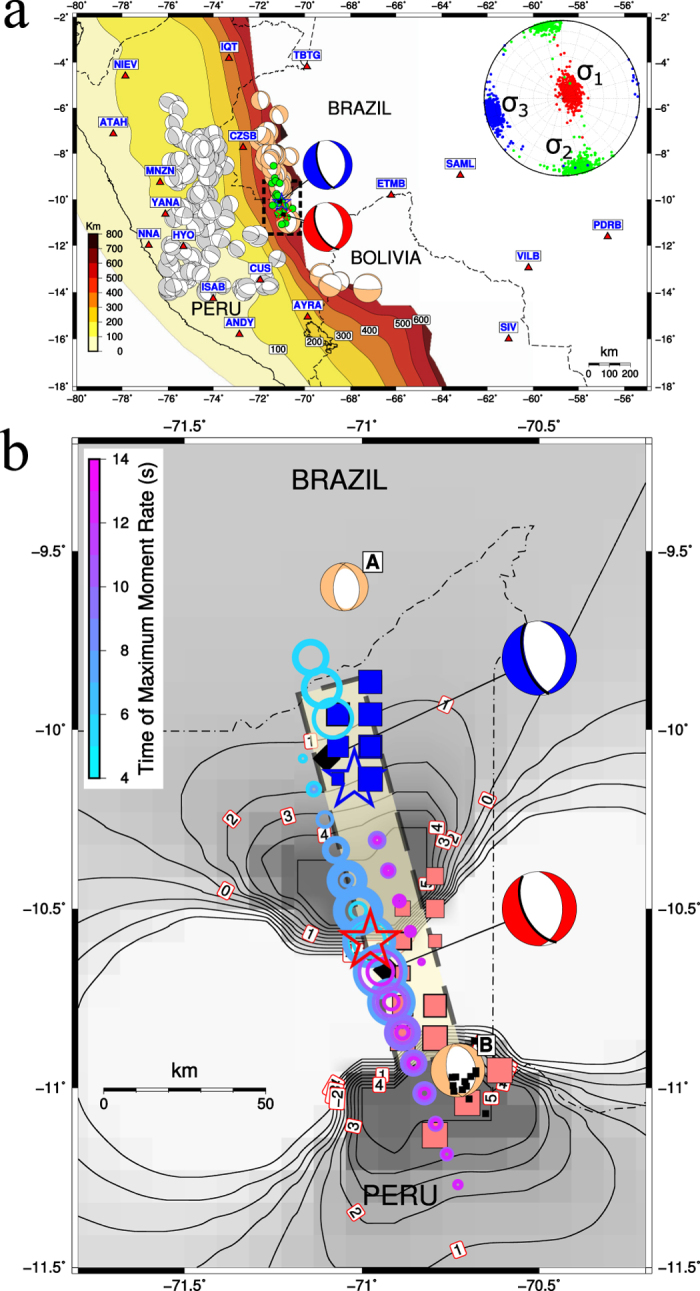
Deep earthquake doublet and Nazca plate subduction. (**a)** Two Mw 7.6 events superimposed onto Slab1.0 isolines and background seismicity (deep fault zone 6°–11°S, light orange beachballs; shallower earthquakes, grey). Regional stress at 600-km depth derived from focal mechanisms (inset); few aftershocks (green circles); seismic stations (triangles). (**b)** Centroids (beachballs), epicentres (stars), and assemblage of possible two-point source models (circles scaled with moment and color-coded by time relative to origin times), with regional back-projection (pink and blue squares), supplemented by rupture stop of Event 1 (small black squares), all fit seismic model (dashed rectangle) filling ∼30-year gap between two neighbouring Mw** **>** **6 events (A and B). Coulomb stress perturbation due to first event (grey-shaded isolines in bars) likely triggered second event. The map was created using software GMT^61^, version 5.2.1, http://gmt.soest.hawaii.edu/projects/gmt/wiki.

On November 24, 2015, two Mw 7.6 earthquakes occurred in the Peru-Brazil region. Event 2 closely followed Event 1, approximately 50** **km northward and 5** **minutes later (Fig. 1 and Table 1), thus forming a doublet, unique in the Peru-Brazil segment. Only a few (∼20) small aftershocks were reported by USGS by the end of November 2015, clearly aligned with the aforementioned 160°-striking fault zone, such paucity of aftershocks being rather typical for warm slabs^14^.

**Figure 2:**
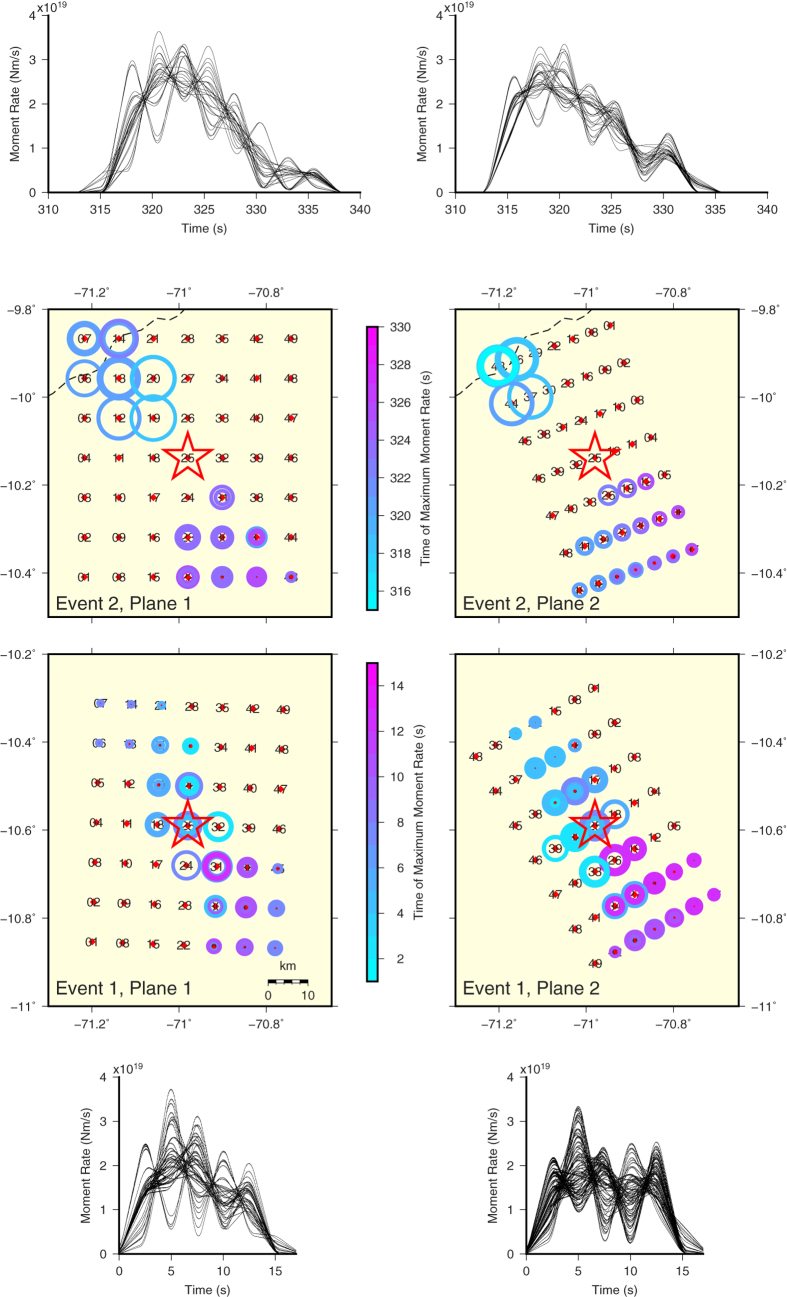
Two-point source models. Waveforms at regional seismic stations (Fig. 1a) were inverted into models of Events 1 and 2. For waveform match between real and synthetic seismograms, see Supplementary Fig. S5. We simultaneously calculated moment-rate time functions (curves) for each source pair in the gridded nodal planes of Table 1 (numbered points), centred at hypocentres (stars). Shown is an assembly of models fitting data almost equally well. The circle radii scale with moment and are color-coded by time, relative to origin times. Robust features are quite clear: late subevents (pink) occurred towards the SE; Event 1 likely consisted of two comparably large subevents, while Event 2 was dominated by a single subevent. For ‘line-source version’ of this modelling, see Fig. 1b.

In contrast to a previous teleseismic study of the doublet^17^, we employed high-quality regional data from recently growing permanent seismic networks to obtain a closer look at the source process. As illustrated in Fig. 1b, we found centroids of Events 1 and 2 (i.e., point-source approximations of the ‘centre of gravity’ of the fault slip) again nicely aligned with the 160°-striking zone. A notably large (>95%) double-couple percentage (i.e., almost pure shear faulting) was clearly demonstrated. Two-point modeling^18^ revealed that the moment-release history of Event 1 likely consisted of two comparably intense episodes (Fig. 2): an early subevent close to the hypocenter, and a later one shifted towards the SE, close to the ‘magic’ azimuth of ∼160°. Total source duration is ∼15** **s, in agreement with the teleseismic study of Event 1 by the SCARDEC method^19^ (reported at http://geoscope.ipgp.fr/).

Moreover, upon seeking to locate a point where the fault rupture terminated^20^, we detected a possible rupture stop of Event 1 at a similar azimuth of 150° (Fig. 1b). Back-projection of regional data confirmed this observation. For details, see Methods and Supplementary Figs S1–4. Thus we understand Event 1 as an almost horizontal unilateral rupture propagation, slab-strike parallel, over a distance of ∼58** **km within 18** **s, yielding an estimated rupture speed Vr** **=** **3.2** **km/s. This regional estimate of Vr, while relatively high, is lower than the teleseismic estimate (4.5** **km/s)^17^, illustrating generally recognized uncertainties in this delicate parameter.

We also modelled waveforms with circular source patches (see Methods). Thus another plausible model of Event 1 appeared, consisting of two equally large circular patches, radius R** **=** **10** **km each, centred at hypocentral distances 10 (the NW patch) and 20** **km (the SE patch). Radial rupture propagation from the hypocentre with constant speed of Vr** **=** **2** **km/s explains the observed total duration of ∼15** **s and is consistent with centroid time, 7** **s after origin (Table 1). This (low-frequency) model provides lower Vr than the rupture stop; nevertheless, in terms of subevents, this model is also unilateral (toward the SE). Even lower speed, such as 1** **km/s, is contraindicated by the relatively large separation of the subevents and relatively small total duration.

Event 2 was somewhat different. It lasted ∼20–25** **s, and it featured a single dominant subevent, displaced from its hypocentre in the direction opposite to Event 1, i.e., towards the NW (Fig. 2). A rupture zone situated essentially northward from the hypocentre of Event 2 was confirmed by the back-projection (Fig. 1b). The dominant part of the rupture process lasted ∼15** **s, well explaining why centroid time (6** **s) was similar to Event 1. The 20-km distance between the centroid and hypocentre then allows an interpretation of the dominant subevent as a circular patch with R = 20** **km, and Vr = 2.7** **km/s, close to the teleseismic estimate^17^.

As a whole, the large variety of possible two-point models in Fig. 2 prevents determination of more specific estimates of Vr, while favouring rupture speeds of 2–3** **km/s. As such, the rupture-to-shear wave-speed ratio 0.36–0.55 rates the doublet as ‘warm’ but with relatively high rupture speeds^14^. Radiated seismic energy for each event has been highly uncertain, (4−5) ± 3 × 10^15^ J.

Several interesting features of the doublet can be confidently resolved, despite these uncertainties. Preferring a robust source characterisation, and preparing for comparison with geodynamic modelling, we ultimately understand Events 1 and 2 as segmented rupture of a single fault (Fig. 1b). In the context of the entire deep fault zone, this fault is striking at ∼160°. Considering that one nodal plane for each event exhibits strike close to 160° (see Planes 2 in Table 1), this plane – dipping towards the SW at ∼60° – is the preferred fault plane. Note that individual analyses of the two events revealed almost no preference for either nodal plane, while here the preference clearly emerges from treatment of the doublet in the context of the neighbouring environment. Furthermore, upon adding information about background seismicity (see Methods), this simplified single-fault model fits into the zone delimited by two Mw6+ events of 1983 and 1990, see A and B in Fig. 1b, respectively. As Mw ≥ 5 earthquakes have not occurred between A and B since 1977, we conclude that the 2015 doublet filled a ∼30-year gap.

Had the two events ruptured the fault without temporal segmentation, the total moment would be equivalent to magnitude Mw 7.8. The question of why the source process was split into two Mw 7.6 events separated by 5** **minutes remains unresolved as a challenge for future study, presumably related to some sort of heterogeneity on the fault inhibiting uniform rupture propagation. Nonetheless, causal relation between the two events seems apparent. Teleseismic back-projections^17^ located an early aftershock of Event 1 (occurring 165 s after nucleating Event 1) near the hypocentre of Event 2. If this represented dynamic triggering of Event 2, then some fault process is needed to explain >2 minutes delay of the complete nucleation of Event 2 (until a measurable seismic signal was radiated). Although such process may possibly have occurred, we propose a more conservative hypothesis of static triggering, because a Coulomb stress change^21^ of 2 bars (0.2 MPa) was induced by Event 1 at the hypocentre of Event 2, as illustrated in Fig. 1b.

Physical understanding of the doublet would be incomplete without viewing these specific events in the light of the regional stress field, derived on the basis of 16 GCMT focal mechanisms (see Methods). Two optimally oriented faults (OOF’s in Table 2) were jointly inverted with the principal stress axes. One OOF exhibits strike 164°, matching that of the entire deep fault zone, providing further support for inferring that the doublet ruptured the nodal planes striking close to ∼160° and dipping ∼60° and documenting the full consistency of the doublet with the regional stress regime. Low fault friction of 0.10–0.25 was also obtained as part of the stress inversion.

As illustrated in the inset to Fig. 1a, the maximum principal stress axis (σ_1_) deviates only slightly (∼10°) from the vertical direction, a feature typical for normal faulting. The intermediate axis (σ_2_) is remarkably well aligned with the 160°-striking deep linear fault, hence parallel to the strike of the slab, and the minimum stress axes is nearly horizontal, perpendicular to the slab. While the vertical σ_1_ axis orientation is characteristic for shallow extensional structures (e.g., rifts), the origin of such a stress state at ∼600 km depth requires some explanation, for which we turn to a geodynamic model of the long-term evolution of regional subduction as governed by the rheological and structural transitions in mantle minerals.

## Geodynamic and Mechanistic Interpretations

**Figure 3:**
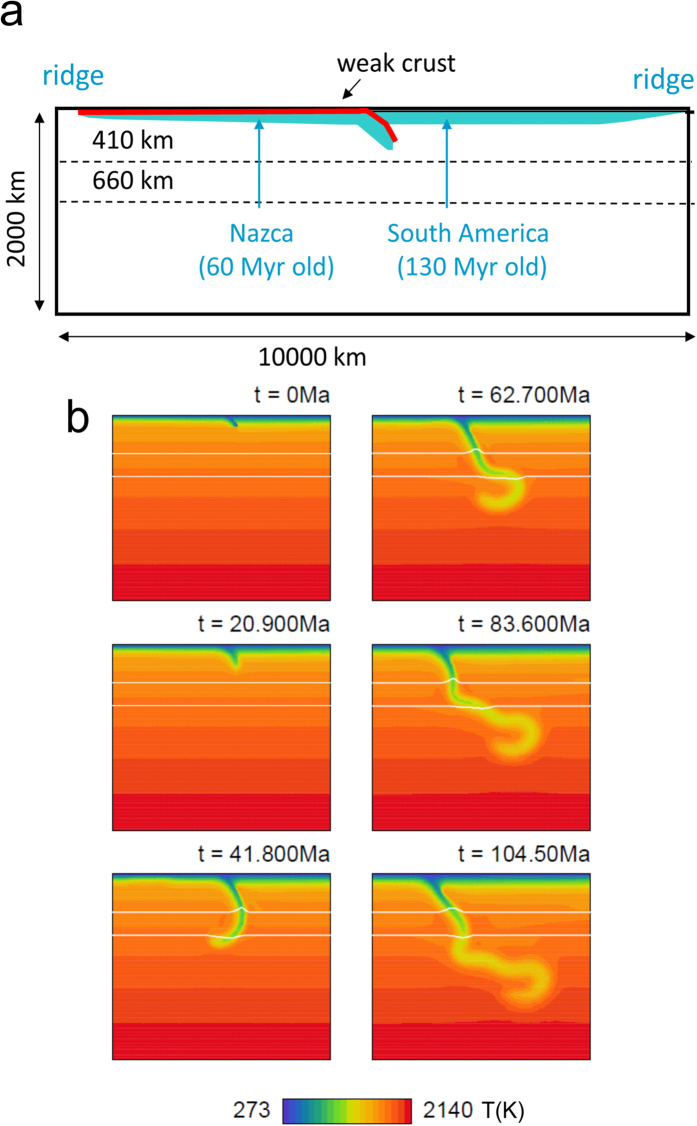
Geodynamic model of Nazca plate evolution. (**a)** Model domain with subducting and overriding plates (blue), and weak crustal decoupling layer (red). (**b)** Temporal evolution of slab temperature; phase-transition boundaries at 410- and 660-km depth (white). Nazca plate subducts slowly, accelerating after ∼30** **Myr due to negative buoyancy of exothermic 410-km transition. Then it slows due to resistance of endothermic 660-km boundary. Stress generated by opposing phase-transition effects weakens slab through power-law stress-limiting deformation mechanism, yielding buckling and trench rollback. Horizontally lying part of slab slowly sinks into lower mantle, reaching ∼1000** **km at ∼105** **Myr.

**Figure 4:**
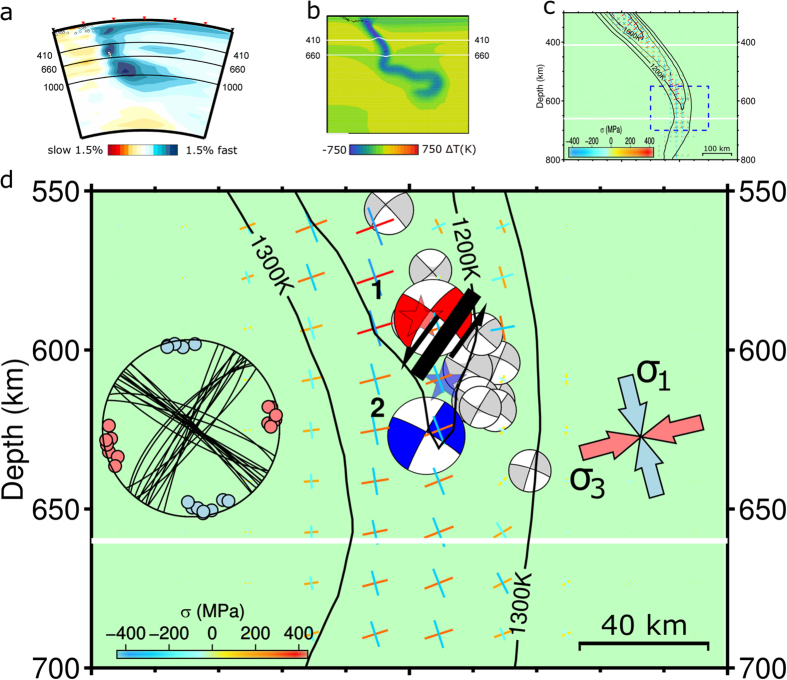
Unified interpretation of seismic data and subduction. (**a)** Seismic tomography at Lat 10°S reveals stagnant horizontal slab^16^ (after their Figure 13 h). (**b)** Geodynamic model of this study agrees with tomography. Colours show temperature anomaly relative to average mantle geotherm. (**c)** Model temperature at earthquake depths of ∼600** **km reaches 1200** **K. (**d)** Principal deviatoric stresses in nearly vertical part of slab align very well with stress directions inferred from focal mechanisms. Model stresses are plotted by color-coded crosses; lengths show absolute values of stress; colours show signed values, negative for compression). Seismic data are shown by nodal planes and P-T axes in left inset and principal stress axes in right inset. Events 1 and 2 ruptured fault schematically shown in centre. Stars and beachballs have same meanings as in Fig. 1.

Our geodynamic interpretation is based on a 2-D Cartesian model of subduction^22^ in which the 60 Myr-old Nazca plate subducts beneath the 130 Myr-old South American plate (see Fig. 3 and Methods). As illustrated in Fig. 4, the slab geometry resulting from model evolution over ∼110** **Myr is consistent with that imaged in seismic tomography^16^. Furthermore, the instantaneous principal deviatoric stresses computed for our geodynamic model in Fig. 4d align very well with those inferred from the moment-tensor inversions: the corresponding maximum compressive axes are oriented nearly vertically but with a slight inclination (∼10°) toward the trench.

The slab is almost vertical in this depth range, and this steeply down-dip orientation of compressive stress axes arises from a combination of viscous forces resisting slab penetration into the lower mantle, buoyancy forces resisting passage across the negative Clapeyron slope of the ringwoodite disproportionation reaction below 660** **km, and deformation associated with slab buckling and stagnation in the transition zone. The geodynamic model offers additional constraint on slab geometry in this region, beyond the general guidance provided by the Slab 1.0 model^15^ based solely upon sparse seismicity.

Another important observation is that the slab is rather warm, due primarily to youth of the subducting lithosphere, with model temperatures reaching at least 1200** **K at the depth of seismicity (Fig. 4d). This calls into question the feasibility of transformational faulting in metastable olivine as a mechanism for rupture initiation, as olivine is very unlikely to persist metastably at such high temperatures^23^. While metastable pyroxene may exhibit greater thermal resilience^24^,^25^, both its low compositional abundance in the slab interior and the generally displacive nature of its structural transitions should limit its ability to nucleate sustained transformational faulting.

On the other hand, an adiabatic thermal instability mechanism is also unlikely, given the apparently high rupture velocity^14^ and the development of a critical state necessary for seismic triggering^26^. Furthermore, Fig. 4d demonstrates (by coincidence of the red star and beachball) that the rupture propagation of Event 1 was essentially parallel to strike of the slab, thus confined to the colder core of the slab, and the same applies for the entire deep fault zone. Such confinement would be consistent with either transformational faulting or dehydration embrittlement but not with thermal instability. Event 2, on the other hand, might have featured some angular difference between rupture propagation and slab strike, thus encountering a broader temperature range while spreading over the fault plane.

## Discussion and Conclusions

Thus, slab thermal state, observed rupture properties, and evident seismic triggering together argue against both transformational faulting and adiabatic thermal instability, leaving a high-pressure variant of the dehydration embrittlement^6^,^27^ hypothesis as the most likely seismogenic mechanism, perhaps associated (within uncertainties in both the thermodynamic model constraining dehydration reactions and our geodynamic model constraining slab temperatures) with dehydration of superhydrous phase B at these depths^28^. At pressures corresponding to such great depths, hydration-dehydration reactions proceed without a positive volume change associated with a fluid phase, yet these reactions yield nanometric crystallisation of product phases which in turn facilitate grain-boundary sliding in a manner similar to crystallisation associated with metastable solid-solid transformational faulting^3^. Such dehydration-mediated deep seismogenesis can also account for the absence of intermediate-depth seismicity in this region, as a bimodal distribution of seismicity with depth arises naturally from the intersection of slab P-T-t paths with various dehydration reactions for slabs only about 100** **K warmer than our model^28^.

Of course, a seismogenic mechanism linked to hydration-dehydration reactions requires the presence of OH at these depths. In significantly warmer portions of the Nazca slab (e.g., in southern Chile), the increased difficulty of transporting significant quantities of hydrated phases below dehydration “choke points” may be contributing to the lack of deep seismicity in these regions. However, it has been argued^29^ that all subducting slabs should be largely dry within the transition zone, based primarily upon laboratory observations of experimental phase-transformation kinetics^30^ and seismological observations attributed to the presence of metastable olivine^31^. On the other hand, it has been argued that slabs must transport a significant amount of water^32^ into the deep transition zone and shallow lower mantle^33^, supported largely by laboratory observations of natural diamond inclusions featuring hydrous ringwoodite from the deep transition zone^34^ and hydrous ferropericlase from the shallow lower mantle^35^, as well as by seismological observations attributed to hydration effects in the transition zone^36^ and to the presence of dehydration-induced melt below 660** **km depth^37^. Despite this ongoing debate, we note, as have others^38^, that patches of dry metastable regions and wet equilibrium regions may be expected to coexist in proximity over a range of spatial scales within complexly faulted, deformed, and hydrothermally altered deep slabs. It thus seems unlikely that hydration-dehydration reactions can be categorically excluded from occurring within all portions of deep slabs.

Nevertheless, an alternative model is available for this particular geographic region. Based on earlier plate tectonic reconstructions, a compound-slab hypothesis has been advanced for Nazca subduction, in which a very cold slab at great depth is attached to a warm one at shallower depths^39^,^40^. If correct, such a model would yield lower temperatures at 600** **km depth than our geodynamic model, thereby perhaps permitting seismogenic transformational faulting while also allowing for the absence of intermediate-depth seismicity. Earlier thermal modelling of such a scenario^41^, however, yielded minimum slab temperatures at ∼600** **km no lower than ∼1000** **K, perhaps still too warm for olivine metastability.

In summary, our results suggest three inferences with regard to plausible seismogenic mechanisms for this deep doublet. High rupture velocities, slab-strike-parallel rupture propagation, and seismic triggering appear to preclude adiabatic thermal instability. High model temperatures cannot support metastable transformational faulting, unless a compound-slab scenario locally juxtaposes warm material at shallow depths and unusually cold material at great depth. Otherwise dehydration-mediated rupture initiation alone remains feasible, as long as some regions of the slab interior remain hydrated as they approach the base of the mantle transition zone.

## Methods

### Seismic data selection

Broadband waveforms provided by IRIS, IMS network (CTBTO), RSBR (Brazilian Seismographic Network) and Seismological Service of Peru were used. Stations with reliable metadata and unclipped records free of instrumental disturbances were selected to provide the best possible azimuthal coverage. These included 18 regional stations (11 Peru, 6 Brazil, 1 Bolivia) at epicentral distances of ∼340–1560** **km, Fig. 1a, whose data were used for most source-parameter calculations. Additional data were obtained from 7 broadband IRIS teleseismic stations between 62° and 72° to constrain the source depth (with error <10 km) using differential pP-P arrival times. Epicentres were determined with formal errors <5 km.

### Location of the rupture stop

Real rupture may radiate high-frequency signals from various points on the fault, e.g., the points where rupture stops^42^. The last stop can be located similarly to the nucleation point if an end of the P-wave group can be determined at a set of stations. We used original, full-band (<5 Hz) instrumentally uncorrected, broad-band records (vertical components), squared, high-passed at 1 Hz, smoothed and normalized. Thus S waves were suppressed, and the records enabled picking the P-group end time. Using point-source synthetics we checked that at most stations the P group is dominated by the direct wave, thus the P-end time refers to the source process, not being biased by propagation effects. Therefore, the difference between the P-end and P-onset is the apparent source duration, which featured a clear azimuthal dependence (Supplementary Fig. S1). The P-end times enabled coherent alignment at 10–12 stations for Event 1, thus providing location of the last rupture stop as shown in Fig. 1b and Table 1. The depth resolution was poor, thus we constrained the location of the rupture stop to the hypocentre depth. The horizontal error was much greater (∼15 km) than the epicentre error (∼5 km), which is why the station elimination (jack-knifing) in Fig. 1b shows a scatter. Coherence of the P-end times for Event 2 was prohibitively low, making location of the rupture stop impossible.

### Back-projection of regional waveforms

A few regional waveforms are generally less suitable for back-projection than massive teleseismic array data. However, thanks to the large source depth (i.e., relatively simple seismograms) and good azimuthal coverage (Fig. 1a), the following technique proved to be useful. Regional velocity waveforms (vertical components at 18 stations), with instrument response removed, were squared, causal band-pass filtered (0.5–2 Hz), smoothed by a 2-s running average, and normalized. Then they were fourth-root stacked over stations and averaged in an 8-s moving time window. The amplitude of the stack (brightness) was recorded as a function of time and position in a horizontal grid at the centroid depth. The results are shown in Fig. 1b, and details can be found in Supplementary Information, Figs S2–4.

### Seismic waveform inversion – point and multiple-point source models

Full-wave Green’s functions were calculated by the discrete-wavenumber method^43^. Velocity model iasp91^44^ was modified for use in Cartesian geometry by the Earth Flattening Approximation^45^, important at epicentral distances >1000 km. Complete regional waveforms were inverted into centroid-moment-tensor (CMT) models and multiple-point-source models using ISOLA software^46^,^47^ (Figs 1b and 2). The CMT (point-source) calculations were made in a low-frequency range 0.02–0.05 Hz, using the least-squares determination of moment tensor and a spatial-temporal grid search. An extended range of 0.02–0.10 Hz was used to obtain multiple-point-source models, applying joint inversion of source pairs^18^, tested also elsewhere^48^,^49^.

### Estimating rupture speed by circular patch models

Determination of spatial extent of the source and rupture speed requires finite-fault modelling. Although real faults have a spatially varying rupture speed, a common practice is to characterize earthquakes by a constant speed Vr. Adopting this simplification, and in order to obtain a Vr estimate as robustly as possible, we performed forward modelling of waveforms at 0.02–0.10 Hz using (discretised) circular patch models. In this method, the focal mechanism is kept fixed, and the slip is radially symmetric and Gaussian distributed along radius R, with its maximum at the patch centre. Rupture propagates from hypocentre, situated either outside or inside the patch. Position of the patch is chosen to reflect the previous two-point source inversions. Waveform fitting yields seismic moment and moment-rate time function. The two parameters (R and Vr) are varied, and the values providing optimal waveform match are preferred. Models whose moment magnitude differs from the previous CMT estimate are rejected. This approach is a simple version of the MuFEx method^50^.

### Establishing background seismicity

We inspected the GCMT and USGS catalogues for occurrence of past events close to the doublet, at Lat −9.5° to −11.0°, Lon −70.5° to −71.5°, deeper than 500 km. The largest Mw 6+ event occurred almost at the centroid of Event 2 in 1977 (Mw 6.1; 1977/04/09). Two nearest Mw 6+ neighbours were reported in 1983 and 1990, north and south of the 2015 doublet (Mw 6.2 of 1983/06/02, and Mw 6.9 of 1990/10/17, see A and B in Fig. 1b, respectively). The nearest Mw 5+ neighbour occurred close to A in 1984 (the Mw 5.9 of 1984/12/24). Only 11 events of 2.5 < mb < 4.9 were reported in the region since 1983. The 2015 doublet occurred in a zone delimited by two significant events of 1983–1984 at the north and one event of 1990 at the south, totally lacking any Mw ≥ 5 earthquake since 1977 (a seismic gap).

### Stress field – seismological estimate

Regional stress field was calculated from the GCMT focal mechanisms of the 16 deep earthquakes of Mw > 6 at Latitude −6° to −12°. The method (code STRESSINVERSE)^51^ returns principal stress axes with their uncertainties (Fig. 1a, inset), and the strike/dip/rake angles of the optimally oriented faults (Table 2). The stress inversion is accompanied by grid search of the friction coefficient which produces the highest overall instability of the investigated faults.

### Geodynamic modelling

We constructed a geodynamic model intended to be representative of the regional subduction environment, similar to prior modelling efforts^52^ but focused on a different goal. In the region of interest, the age of subducting lithosphere at the trench is estimated currently to be 50–55 Ma but has varied over the range 50–70 Ma during the past 80 Myr^52^,^53^, with subduction having continued over approximately 100 Myr.

Buoyancy-driven flow in Earth’s mantle is governed by the set of equations including continuity, momentum, thermal, constitutive and state equations. Here we use the extended Boussinesq approximation without internal heating and solve the system by the finite-element method using the package SEPRAN^54^.

We employ a 2D Cartesian model^22^ with setup shown in Fig. 3a. Impermeable free slip is prescribed on all boundaries. The subducting plate stretches from the ridge in the upper left corner to the trench located in the middle of the upper boundary. Another ridge, associated with the overriding plate, is positioned in the upper right corner and ensures horizontal mobility of the overriding plate. The subducting plate is covered by a 10-km-thick crust-like layer. The low viscosity of this layer lowers friction at the contact of the plates and facilitates subduction^55^. The position of this weak layer is tracked using particle tracers, and at the depth of 300 km it is replaced by mantle material for numerical convenience. No intrinsic buoyancy anomaly is associated with this crust.

Although the complex 3-D evolution of Nazca subduction in this region, involving changes in convergence direction, migrating ridges, and subduction of volcanic edifices, cannot be fully captured in a simplified 2-D model, we can explore the primary controlling mechanisms and evaluate the first-order dynamical consequences. In our 2-D model, initial temperature distribution in the subducting plate follows a half-space model with increasing lithospheric age, reaching 60 Myr at the trench. A stripe of a younger lithosphere (∼10 Myr old), 400 km wide and initially located 1000 km to the left from the trench, is initially prescribed on the subducting plate to ensure lithospheric age of ∼60 Myr at trench at time ∼50 Ma, in accord with tectonic reconstructions. The overriding plate that starts from the upper right ridge also follows a half-space model, with an age of 130 Myr at the trench. Below the lithosphere, initial temperature follows an adiabat with potential temperature of 1573 K. Thermal boundary conditions prescribe constant temperature at the top and bottom and zero heat flux at the sides.

Our nonlinear composite rheology combines diffusion creep, dislocation creep and a power-law stress limiter (approximating Peierls creep) in the upper mantle^56^. Effective viscosity is calculated from viscosities of the individual creep mechanisms. In the lower mantle, dislocation creep is assumed to play only a minor role; we therefore assume that it deforms solely by diffusion creep. Activation parameters in the upper mantle are based on wet olivine rheology^57^. Lower mantle activation parameters are based on slab sinking-speed analysis^58^. The transition between upper and lower mantle rheology is smoothed over the uppermost 300 km of the lower mantle, so that a lower mantle viscosity of 2.2 × 10^22^ Pas is reached at the depth of 1000 km.

Our assumed thermal expansivity decreases with depth from 3 × 10^−5^ K^−1^ at the surface to 1.2 × 10^−5^ K^−1^ at a depth of 2000 km^59^,^60^. Thermal diffusivity is constant. Major mantle phase transitions at 410-km and 660-km depths are included using harmonic parameterisation of a phase function with respective Clapeyron slopes of 2 MPa/K at 410 km and −1.5 MPa/K at 660 km.

Temporal evolution of the system is illustrated in Fig. 3b.

### Code availability

The software package ISOLA (authors E. Sokos and J. Zahradník), used for most of seismic analyses, can be accessed at http://geo.mff.cuni.cz/∼jz/for_Costa_Rica/.

## Additional Information

**How to cite this article:** Zahradník, J. *et al*. A recent deep earthquake doublet in light of long-term evolution of Nazca subduction. *Sci. Rep.* **7**, 45153; doi: 10.1038/srep45153 (2017).

**Publisher's note:** Springer Nature remains neutral with regard to jurisdictional claims in published maps and institutional affiliations.

## Supplementary Material

Supplementary Information

## Figures and Tables

**Table 1:** Basic parameters of the studied doublet of November 24, 2015.

	Event	Time	Lat N (°)	Lon E (°)	Depth (km)					
**Hypocentre**	1	22:45:38	−10.5893	−70.9768	590					
	2	22:50:53	−10.1403	−71.0220	610					
	**Event**	**Time**	**Lat N (°)**	**Lon E (°)**	**Depth (km)**	Lapse Time* (s)	Distance* (km)			
**Rupture Stop**	1	22:45:56	−10.99	−70.71	590	18	58			
	**Event**	**Time**	**Lat N (°)**	**Lon E (°)**	**Depth (km)**	Plane 1** (s/d/r)	Plane 2** (s/d/r)	**DC (%)**	**Mo (Nm)**	**Mw**
**Centroid MT**	1	22:45:45	−10.67	−70.93	590	2/40/−64	150/55/−110	96	2.7 × 10^20^	7.6
	2	22:50:59	−10.08	−71.10	627	1/30/−73	162/61/−99	100	3.1 × 10^20^	7.6

*Lapse Time and Distance are calculated with respect to Hypocentre. **s/d/r denotes strike, dip, and rake angles (°).

**Table 2:** Principal axes of the regional stress field and the optimally oriented faults (OOFs), derived from 16 GCMT focal mechanisms.

Stress axis	Azimuth (°)	Plunge (°)	
σ_1_	61	77	
σ_2_	165	3	
σ_3_	256	13	
**OOFs**	**Strike (°)**	**Dip (°)**	**Rake (°)**
1	349	39	−85
2	164	65	−93
