# Supplementary material for: A recent deep earthquake doublet in light of long-term evolution of Nazca subduction
Source: Sci Rep. 2017 Mar 31;7:45153. doi: 10.1038/srep45153 (PMC5374489; doi:10.1038/srep45153)
Supplement: Supplementary Information [file srep45153-s1.pdf]

Supplementary Information

A recent deep earthquake doublet in light of long-term evolution of Nazca subduction

J. Zahradník[1)] [*], H. Čížková[1)], C. Bina[2)],

E. Sokos[3)], J. Janský[1)] , H. Tavera[4)], J. Carvalho[5)]

[1)] Charles University, Prague, Czech Republic

[2)] Northwestern University, U.S.A.

[3)] University of Patras, Greece

[4)] Geophysical Institute of Peru, Lima, Peru

[5)] Seismological Observatory, University of Brasilia, Brazil

Directivity of Event 1

Position of hypocentre, centroid and the last rupture stop indicated that Event 1 was predominantly behaving as unilateral rupture. This is further corroborated here by inspecting directivity. At most of the regional stations used, the P-wave group is dominated by the direct wave (as checked by calculating a point-source impulse response); hence the apparent source duration is directly estimated by subtracting hypocentre time from the time of the P-wave end. In Fig. S1, we plot the apparent duration as a function of the station azimuth. For Event 1 the last rupture stop was located at a distance of L = 58 km from hypocentre, with an azimuth of 150°. Therefore, in Fig. S1, we compare the observed duration with the duration predicted for such a unilateral model, assuming a horizontally propagating rupture. The only least-square fitted parameter is L/Vr (where Vr is the rupture speed), linearly related with the apparent duration, from which we obtain rupture speed Vr=3.0. The corresponding source duration of 19 s is consistent with the two-point source models. Note that similar (south-east) rupture propagation has been suggested for another deep event of the investigated region, Mw 6.8 of June 20, 2003[1].

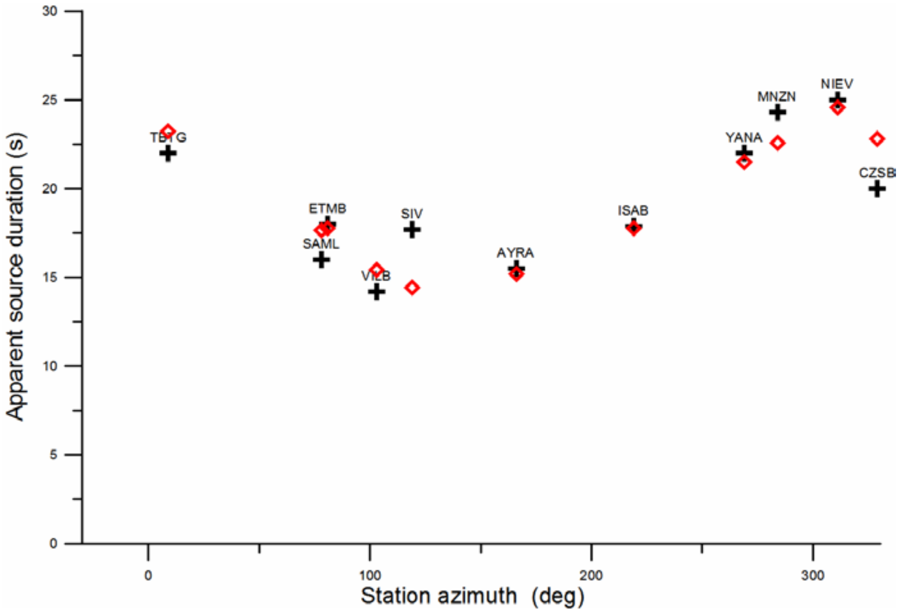

Figure S1. Directivity of Event 1. The azimuthal variation of the apparent source duration (crosses) is in agreement with a unilateral model (diamonds).

Back-projection of synthetics

Before back-projecting real data we make a test of station coverage, following prior work[2] (their section 7). A point-source event is supposed to have its epicentre at point (0,0) in a NS-EW grid. Fictitious stations are situated at the same position as 18 real regional stations of this paper. Also the velocity model is the same. The assumed source depth is 620 km. Two experiments are made, in which we back-project synthetic data onto a horizontal grid situated at the true source depth, Fig. S2a,b,c and also at an erroneous depth (20 km shallower), Fig. S2d. A temporal error is supposed.

In Fig. S2a we assume non-realistically accurate data (error +/- 0.5 s); this option makes the station strips quite narrow and enables us to see that the real station coverage is very good. When increasing the temporal error to +/- 3s (in Fig. S2b), the brightness pattern is smoothed and the point source is imaged as an ellipse, ~100 km long, elongated in the SSW-NNE direction (Fig. S2b); this is caused by a small imperfection of the coverage. Nevertheless, the image remains centred at the true epicentre. When the error is +/- 1 s, and the back-projection is made at the correct depth (620 km), the source image is good (Fig. S2c), but when we perform back-projection at a grid situated at the depth of 600 km, the same temporal error yields an important distortion (Fig. S2d): the image splits into a major and a minor patch, neither of them having the correct position (0,0).

When projecting real data at regional distances, the signal coherence across stations is low, hence a smoothing is necessary; this is equivalent to a larger temporal error in these tests. Therefore, we must expect blurred images of point sources. Moreover, the brightness maxima might be spatially biased due to incorrect depth. Naturally, finite sources bring even more complications.

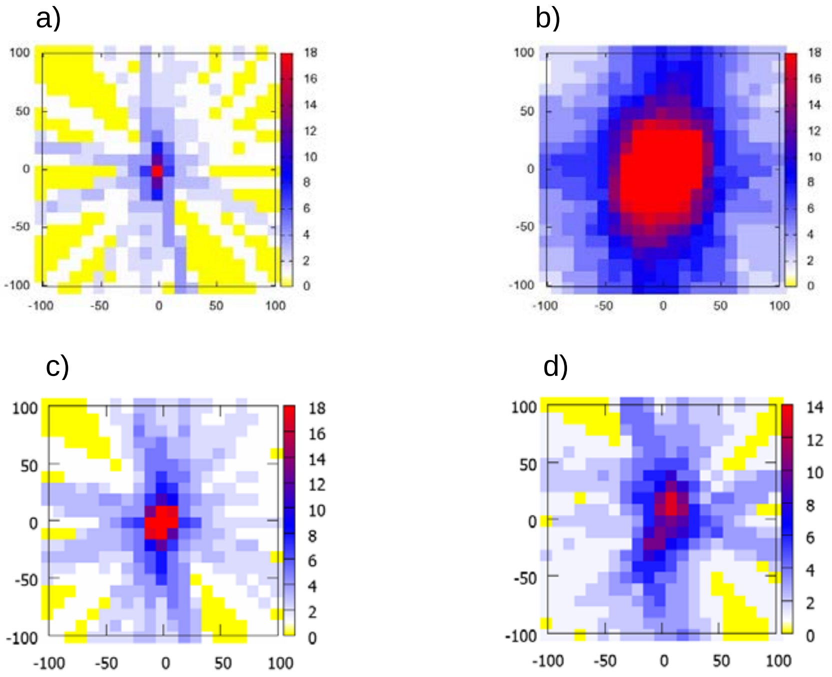

Figure S2. Synthetic modelling the back-projection for a point source in a NS-EW, 200x200 km grid. In panels **a)** and **b)** the back-projection makes use of the correct source depth, assuming temporal error of +/- 0.5 and +/- 3 seconds, respectively. In panels **c)** and **d)** the back-projection assumes a temporal error of +/- 1 s and makes use of correct depth in panel c and an incorrect depth (20 km shallower) in panel d.

Back-projection of Event 1

Regional velocity waveforms (vertical components at 18 stations), with instrument response removed, were squared, causal band-pass filtered (0.5-2 Hz), smoothed by a 2-s running average, and normalized. Then they were fourth-root stacked over stations and averaged in an 8-s moving time window. The amplitude of the stack (brightness) was recorded as a function of time and position in a horizontal grid at the centroid depth. Before this procedure, first arrivals were picked and their alignment at the hypocentre position was optimized by adaptive stacking,

using code TCAS[3], thus providing station corrections. The frequency range and smoothing parameters were varied to obtain stable results.

The results are provided in Fig. S3. Note that the individual brightness maxima (or bright spots), in Fig. S3e apparently occur near the hypocentre not only very early but also very late (0 and 20 s after origin time). The individual bright spots thus cannot be used to infer the rupture-speed value. Only the overall pattern of Fig. S3e is meaningful. In Fig. S3f we present all brightness spots within a 95%-100% range of the maximum brightness, at each time slice. The obtained spatial pattern is stable (smooth) thanks to the 8-s stacking window. Time-integrated pattern (Fig. S3g), similar to Fig. S3e, confirms that the source process was unilateral (SSE of hypocentre), lasting 20 s and spanning distance ~60 km, thus providing a very rough estimate of rupture speed Vr~3 km/s. Panel S3e is included in the final seismic interpretation of the doublet (Fig. 1b of the main text) with omission of the (color-coded) temporal information.

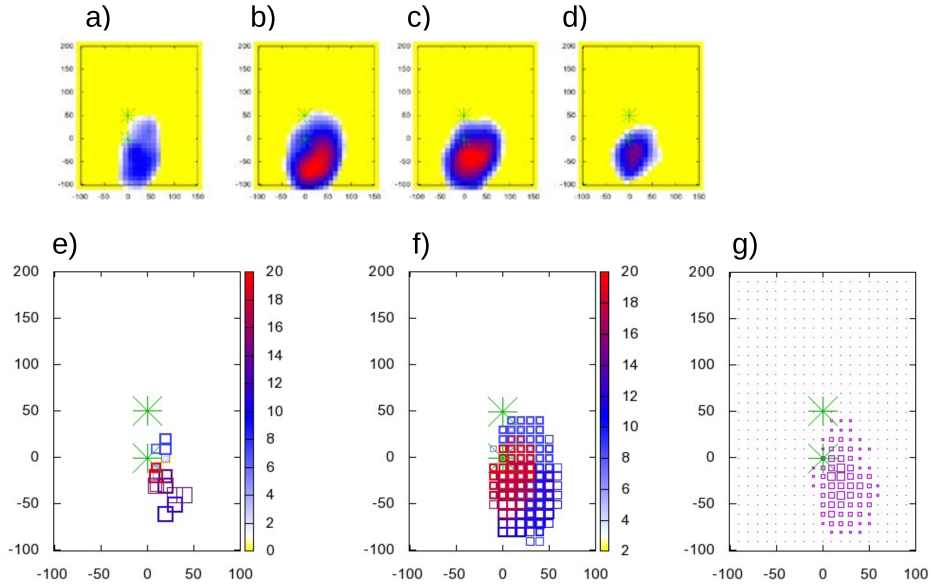

Figure S3. Back-projection of regional waveforms of Event 1. Panels **a) - d)** show the temporal evolution of the brightness in a NS-EW kilometric grid at 10, 12, 14 and 16 s after hypocentre time, respectively. Colour represents the brightness (beam power) in arbitrary units, using same scale in all four panels. Epicentres of Event 1 and 2 are shown by green asterisks. Panel **e)** demonstrates the maximum brightness values Bmax over the grid at every calculated time slice (80 slices, covering 20 seconds after origin time, the increment of 0.25 s); the symbols are

scaled with brightness value and color-coded with time. Panel **f)** is obtained by plotting, at each time, the brightness between 0.95 x Bmax and Bmax. Panel **g)** is the same as in panel f) but time-integrated 20 seconds after origin time. The symbol size scale (brightness) in panels e) to g) is not the same.

Back-projection of Event 2

The same technique as used for Event 1 was applied to Event 2 (Fig. S4). The spatial extent of the brightness maxima in Fig. S4e is comparable to Event 1, or even larger. The figures seem to indicate unilateral rupture propagation toward the NE. However, when comparing with the synthetic test in Fig. S2d, it appears possible that the obtained pattern is affected by the inclination of the fault plane. This is why we put a question mark on the NE patch in Fig. S4e; it could be an artefact, because centroid of Event 2 is situated 20 km NW of hypocentre, not toward the NE.

The hypothesis that the NE patch is an artefact has been supported also by an additional test in which we back-projected seismograms on the nodal planes, instead of the horizontal planes. In that case we observed instability of Event 2; indeed, the bright spot was artificially 'moving' up-dip with progressive time. No instability like that was observed in Event 1. It is likely that Event 2 involved more non-horizontal rupture evolution than Event 1, but such a non-horizontal evolution is hardly resolvable.

For all these reasons the NE patch (marked by the question mark) is not included in the final seismic interpretation of the doublet in Fig. 1b of the main text.

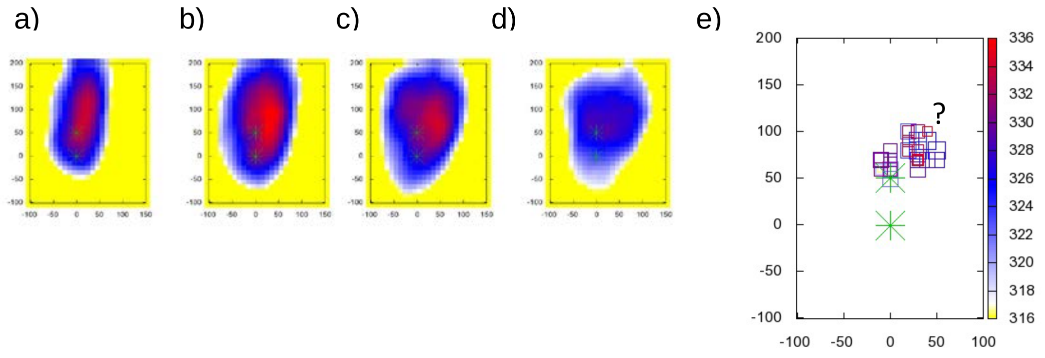

Figure. S4. Back-projection of regional waveforms of Event 2. Analogous to Fig. S3. Panels **a)** - **d)** show the temporal evolution of the brightness in a NS-EW kilometric grid at 8, 10, 12, and 14 s after hypocentre time, respectively. Epicentres of Event 1 and 2 are shown by asterisks. **e)** Maximum brightness Bmax at each computed time slice; time is measured with respect to origin time of Event 1 (time 0). The question mark shows limited reliability of the NE patch.

Two-point source modelling

We invert waveforms for two point-source subevents simultaneously[4]. Their focal mechanisms and the total scalar moment of each trial source pair is pre-constrained, according to the centroid-moment-tensor solution. The method systematically inspects all existing pairs in a grid of trial source positions. For each pair, the moment-rate time functions are jointly calculated for both subevents. The time functions are parametrized by isosceles triangles of a constant duration and shift. After preliminary tests, the moment-rate time function for each member of any source pair was represented by 12 isosceles triangles of 5-s duration, overlapped with each other by 2.5 s. Their relative weights are calculated by the non-negative least-squares method[5]. Therefore, for brevity, the whole technique is denoted NNLS. With NNLS we obtain a suite of solutions that match the data within a chosen threshold of variance reduction, thus obtaining certain insight into the model uncertainty. Note that our multiple-point-source models do not require any assumption about rupture speed. An example of a typical waveform match for two-point models of Fig. 2 in the main text (variance reduction of 0.7) is shown in Fig. S5.

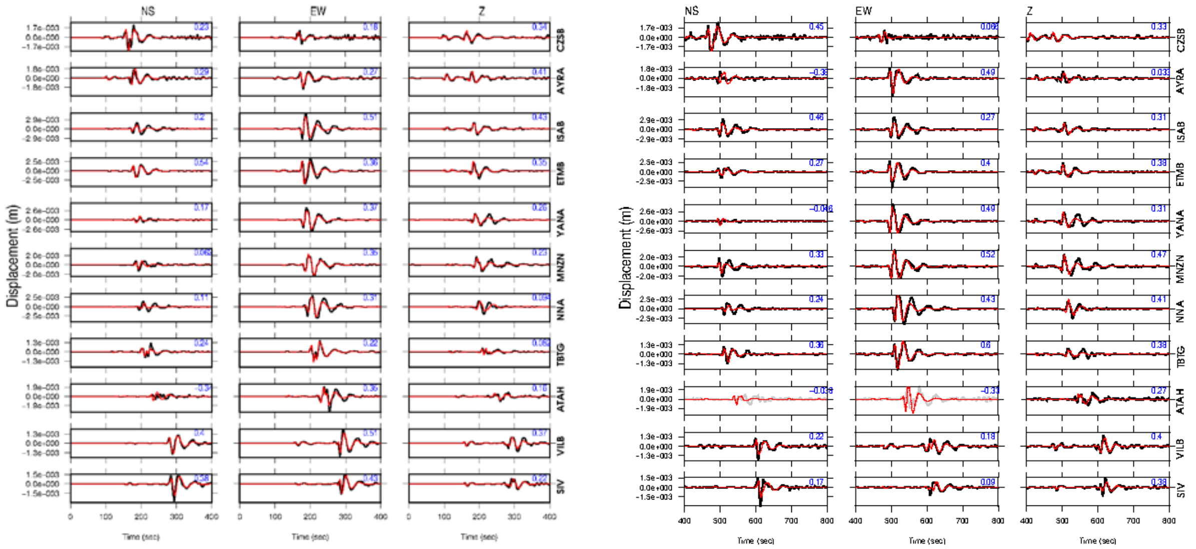

Figure S5. A typical waveform match between real (black) and synthetic (red) displacement waveforms in the frequency range 0.02-0.10 Hz: **Left)** Event 1, **Right)** Event 2, both at Plane 2 (Table 1 of the main text). The station codes appear to the right; for the station map see Fig. 1a of the main text. The NS and EW components of ATAH station were removed from inversion of Event 2 due to instrumental disturbance. Time 0 is the origin time of Event 1.

References for Supplementary Information

1. Tavera, H., Manrique, M., Salas, H. & Fernández, E. Análisis del mecanismo del sismo de foco profundo del 20 de Junio de 2003 (límite Perú-Brasil). *Boletín la Soc. Geológica del Perú* **96,** 87–94 (2003).

2. Zahradník, J., Janský, J. & Plicka, V. Analysis of the source scanning algorithm with a new P-wave picker. *J. Seismol.* **19,** 423–441 (2015).

3. Rawlinson, N. & Kennett, B. L. N. Rapid estimation of relative and absolute delay times across a network by adaptive stacking. *Geophys. J. Int.* **157,** 332–340 (2004).

4. Zahradnik, J. & Sokos, E. The Mw 7.1 Van, Eastern Turkey, earthquake 2011: two-point source modelling by iterative deconvolution and non-negative least squares. *Geophys. J. Int.* **196,** 522–538 (2014).

5. Lawson, C. & Hanson, R. *Solving Least Squares Problems*. (Prentice-Hall, 1974).
